# Pulmonary Tuberculosis After Gastric Bypass: A Very Rare Complication

**DOI:** 10.7759/cureus.3108

**Published:** 2018-08-06

**Authors:** Muhammad Israr Ul Haq, Usama Talib, Armghan H Ans, Umer Razzaq, Hassan Mehmood

**Affiliations:** 1 Surgery, New York University Langone Medical Center, New York, USA; 2 Internal Medicine, North Shore Salem Hospital, Salem, USA; 3 Cardiology, University of Pennsylvania, Philadelphia, USA; 4 Internal Medicine, University of Nevada, Nevada, USA; 5 Internal Medicine, Temple University/Conemaugh Medical Center, Johnstown, USA

**Keywords:** tuberculosis, gastric bypass complications, rygb

## Abstract

Laparoscopic Roux-en-Y gastric bypass (RYGB) is the most commonly performed bariatric surgical procedure with successful outcomes. RYGB has multiple positive outcomes, including sustained weight reduction, resolution of co-morbidities and improvement in the overall health. RYGB has many complications like any other surgery, but the development of tuberculosis (TB) either pulmonary or extra-pulmonary secondary to RYGB is very rare. We present a 32-year-old female with the history of a successful RYGB three years ago, who presented with signs and symptoms of possible TB which was later confirmed with sputum acid-fast bacilli and sputum culture. She was treated with anti-tuberculosis treatment (ATT) drugs for six months with complete resolution of her symptoms. We recommend raising awareness in the health care professionals about this rare complication of RYGB in the need of time.

## Introduction

Roux-en-Y gastric bypass (RYGB) is the most commonly performed bariatric surgical procedures with successful outcomes. RYGB has multiple positive outcomes, including sustained weight reduction, resolution of co-morbidities and improvement in the overall health. RYGB is associated with some complications such as infection and perforation leading to catastrophic leakage. Apart from these conventional complications, RYGB predisposes these patients to tuberculosis (TB), which can be pulmonary or extrapulmonary [1,2]. The risk of TB in gastric bypass patients is higher than that in the general population [3]. The exact incidence of TB in RYGB is unknown and very few cases have been reported so far. According to one study, patients who underwent RYGB for morbid obesity have a 60-fold higher incidence of pulmonary or extrapulmonary TB than the general population [4].

## Case presentation

**Figure 1:**
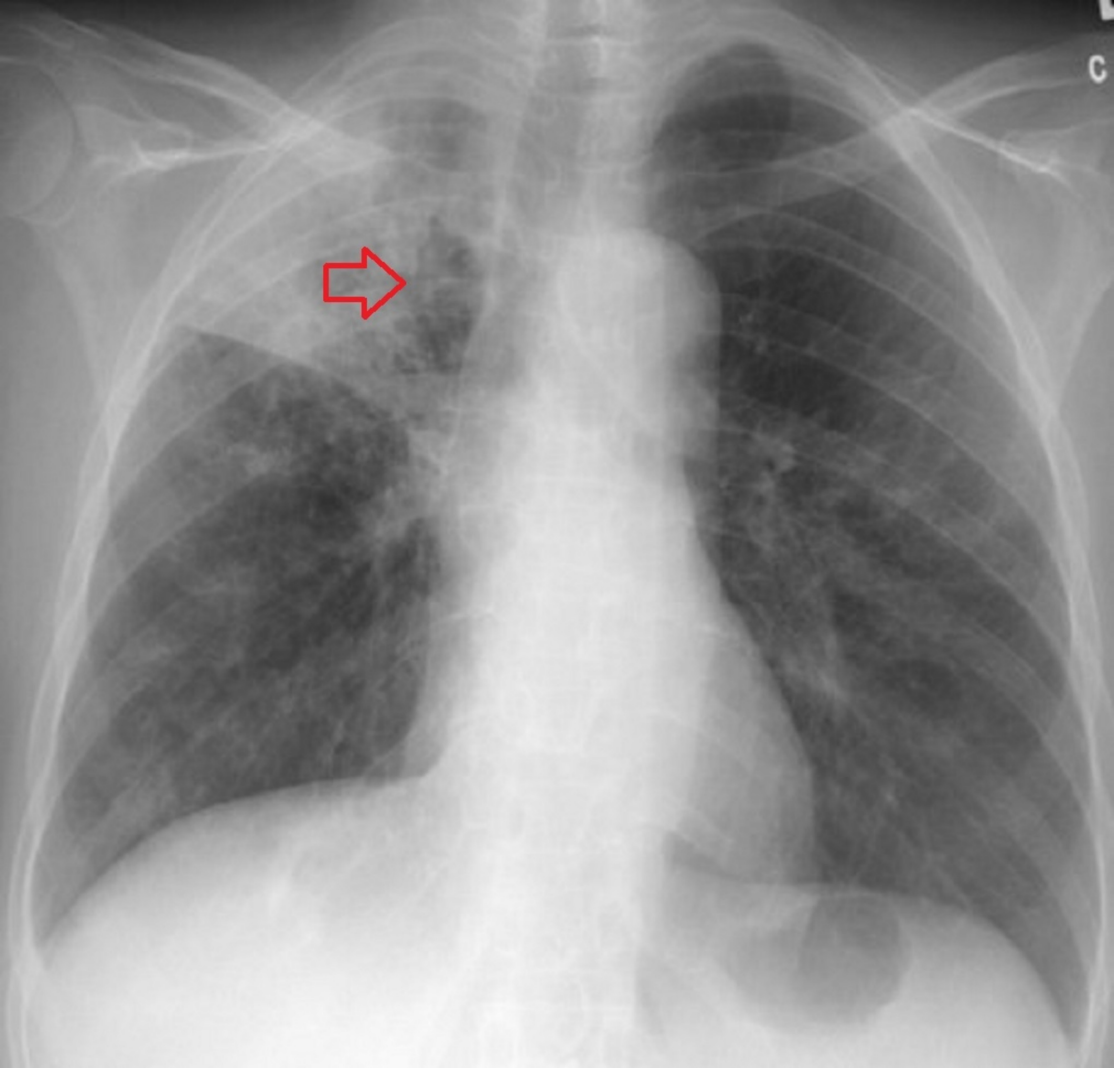
X-ray tuberculosis. Probable cavity in the right lung apex with evidence of consolidation.

**Figure 2:**
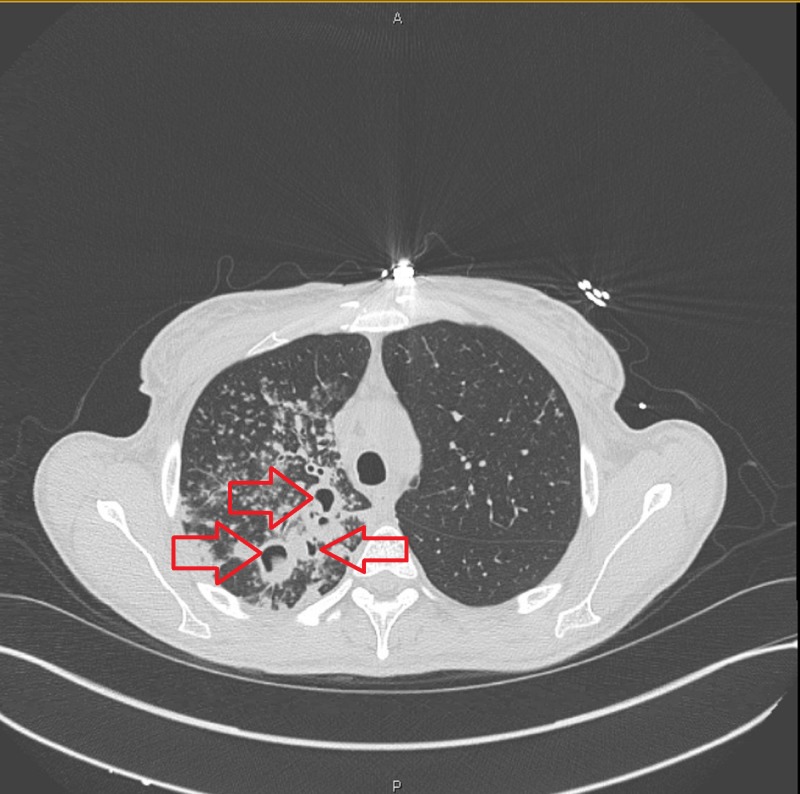
Chest computed tomography of the patient with tuberculosis. Multiple cavities surrounded by patches of consolidation with some traction bronchiectasis.

**Figure 3:**
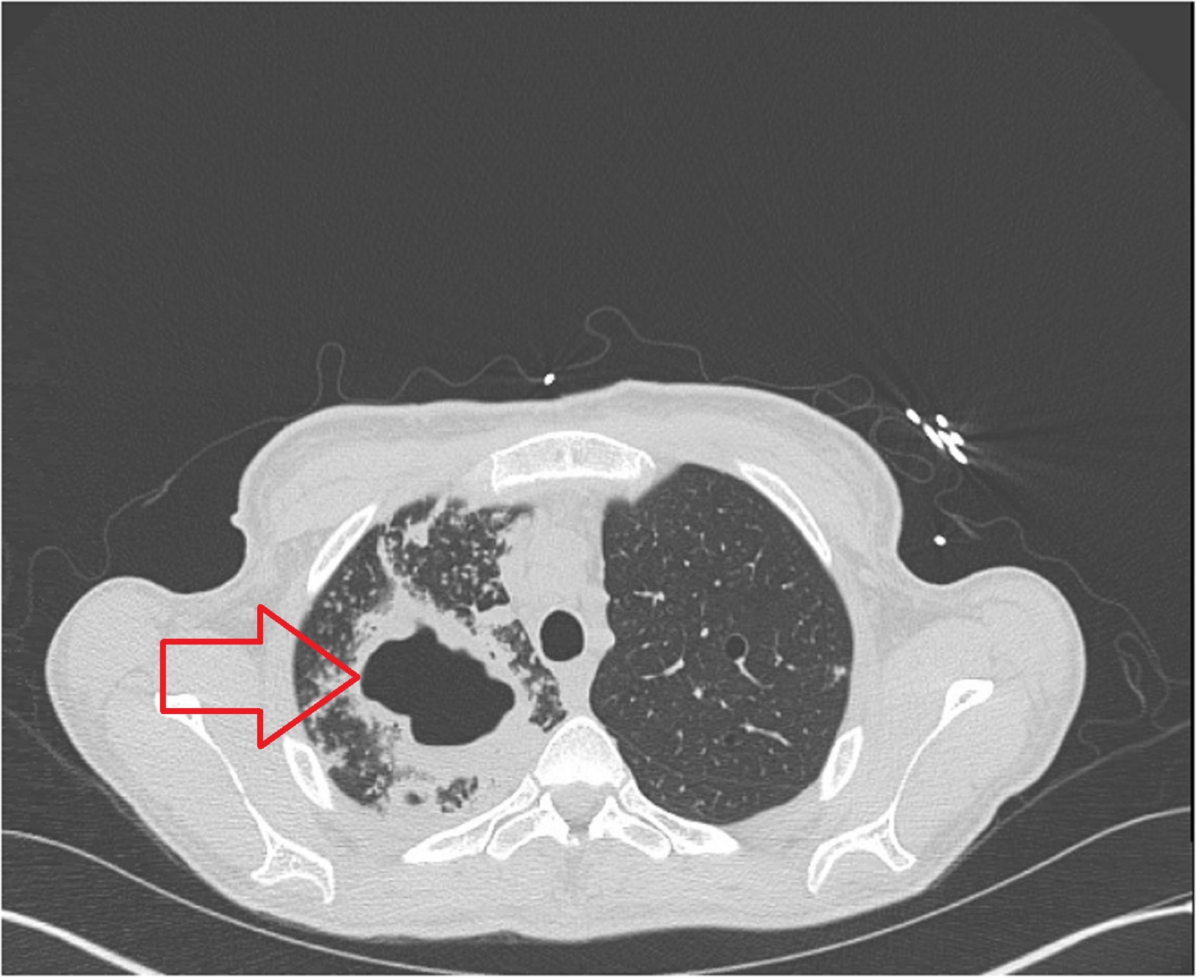
Computed tomography of the patient with tuberculosis. Large cavitation with surrounding area of consolidation.

A 32-year-old female with history of diabetes mellitus type one and a successful RYGB for morbid obesity three years ago presented to our clinic with the complaints of cough, greenish yellow sputum production, chills and night sweats for the last three months. She was feeling more fatigued, generalized weakness and unintentionally lost 33 pounds during that time. She denied recent history of travel out of state. The patient was in the United States and had never been to a country with endemic tuberculosis. She denied recent remote history of incarceration. She also denied hemoptysis, shortness of breath, headache or fever. She had no history of being diagnosed with TB or history of recent sick contacts. On physical examination, her temperature was 36.9°C, blood pressure was 116/60 mm Hg, pulse was 84 per minute and respiratory rate was 16 per minute. On chest auscultation, few rhonchi were present in the right upper lung and the rest of the physical examination was unremarkable. The Initial blood work showed sodium of 134 mmol/L (136–145 mmol/L), potassium of 4.2 mmol/L (3.5–5.1 mmol/L), bicarbonate of 28 mEq/L (23–31 mEq/L), blood urea nitrogen (BUN) of 6 mg/dL (9–21 mg/dL), creatinine of 0.33 mg/dL (0.6–1.1 mg/dL), glucose of 150 mg/dL (80–115 mg/dL) and liver function tests were within normal range. Her white blood cell count was 14,500/µL (4500–11000/µL) with 81% neutrophils. Her chest X-ray showed multiple small nodular opacities throughout the right lung with a probable cavity in the right lung apex (Figure 1). Suspicion was raised for possible active TB and she was admitted in airborne isolation. Two peripheral blood cultures, sputum culture along with acid-fast bacilli (AFB) were ordered. Computed tomography (CT) chest without contrast showed multiple thick-walled cavities in the right upper lobe. The largest cavity was 7 cm. There were innumerable centrilobular nodules and tree in bud opacities throughout the right lung (Figures 2, 3). Mycobacterium tuberculosis bacillus was detected by sputum AFB staining and later confirmed by culture and polymerase chain reaction (PCR) assay. She also had a positive QuantiFERON test. She was started on anti-tuberculosis treatment (ATT) medications including Isoniazid 300 mg daily, Rifampin 300 mg twice daily, Ethambutol 800 mg daily, Pyrazinamide 1000 mg daily and Pyridoxine 50 mg daily for the first two months and then two drugs Isoniazid and Rifampin for the next four months. After three days of hospital stay, she started showing some improvement in her symptoms such as cough and night sweats. She understood the isolation precautions, especially respiratory precautions and use of mask in the presence of other individuals. She was later discharged in stable condition with the above-mentioned medications. She had complete resolution of her symptoms in two months and completed six months course of ATT.

## Discussion

Pulmonary and extrapulmonary TB is understood to be a common entity after gastric resection [5]. RYGB is a procedure that may confer a similar risk. The mechanism is not completely understood, but may be related to loss of gastric acidity; however, the risk of TB among individuals with gastric achlorhydria has not been studied. In postoperative period after gastric bypass, zinc and protein deficiency may be the probable reason of increased incidence of TB and both conditions have been reported to adversely affect cell-mediated immune responses [6-9]. According to one study, patients who underwent RYGB for morbid obesity have 60-fold higher incidence of pulmonary or extrapulmonary TB than general population [4]. Jejunoileal bypass has become less popular due to a higher incidence of complications and availability of alternative surgical procedures for morbid obesity. According to one study, 14 cases of pulmonary TB were observed in patients who underwent jejunoileal bypass [10].

More people are exposed to TB in developing countries as compared to developed countries; thus, the risk of acquiring TB in individuals who underwent weight loss surgery, is higher. A detailed history of exposure can help in minimizing the reactivation after gastric bypass surgery. Mantoux tests with five tuberculin unit can be performed before the surgery. The results of this test must be interpreted carefully. The induration of 5 mm or more is considered positive in human immunodeficiency virus (HIV)-positive person, recent contacts of active TB cases, persons with nodular or fibrotic changes on chest X-ray consistent with previously healed TB, organ transplant recipients and other immunosuppressed patients, and end-stage renal disease. The induration of 10 mm or more is considered positive in recent arrivals (less than five years) from high-prevalence countries, injectable drug users, residents and employees of high-risk congregate settings (e.g., prisons, nursing homes, hospitals, homeless shelters), mycobacteriology lab personnel, persons with clinical conditions that place them at high risk (e.g., diabetes, prolonged corticosteroid therapy, leukemia, end-stage renal disease, chronic malabsorption syndromes, low body weight), children less than four years of age, or infants, children and adolescents exposed to adults in high-risk categories. The induration of 15 mm or more is considered to be positive in persons with no known risk factors for tuberculosis [11].

The clinical manifestations of pulmonary TB include fever, fatigue, cough with sputum production, night sweats, arthralgia, anorexia, weight loss and pharyngitis. Chest radiograph can show upper and lower lobe infiltrates along with hilar adenopathy. Symptoms may be acute in onset and can be confused with bacterial pneumonia, and asthma. The clinical manifestations can also be subacute or chronic, resembling bronchogenic carcinoma [12].

The definitive diagnosis of pulmonary TB requires a detailed history, physical examination, radiographic imaging and laboratory studies. Chest radiography is the part of initial approach due to its easy availability and cost. Chest computed tomography is more sensitive than plain chest radiography for identifying early pulmonary tuberculosis. Laboratory testing includes sputum AFB smear, mycobacterial culture, and molecular tests. The detection of AFB on microscopic examination of stained sputum smears is the most rapid and inexpensive TB diagnostic tool. All clinical specimens suspected of containing mycobacteria should be cultured. Molecular methods are also available like nucleic acid amplification (NAA) tests.

The recommended treatment for pulmonary TB is conventional ATT therapy for a minimum of six months. There are two phases of treatment, i.e., intensive phase and continuation phase. The intensive phase usually consists of four drugs, i.e., Isoniazid, Rifampin, Pyrazinamide, and Ethambutol administered for two months. The continuation phase (regimen beyond the first two months) usually consists of two drugs, Isoniazid and Rifampin administered for four additional months, for a total of six months. If the patient does not respond clinically, the therapeutic drugs might be poorly absorbed. Their levels should be measured and their doses adjusted accordingly. Our patient responded quickly to ATT therapy, we believe our patient had no problem with medication absorption. Drug-resistant TB should be suspected in the setting of relevant risk factors which includes exposure to an individual with known or suspected drug-resistant TB, residence in or travel to a region with high prevalence of drug-resistant tuberculosis, residence in or work in an institution or setting with documented drug-resistant tuberculosis, among foreign-born individuals: Emigration within the previous two years. We did not test drug resistance as our patient had none of these risk factors.

## Conclusions

RYGB is a complex procedure with a certain degree of understood malabsorption to improve surgical outcome in the form of weight loss. RYGB is associated with some complications including the development of TB. Though pulmonary or extrapulmonary TB is very rare, high degree of suspicion and early diagnosis is important for better prognosis. Our patient never had Mantoux test, however, it should be performed preoperatively on all potential RYGB candidates. A positive test may be followed by an X-ray to differentiate active versus latent TB. Any atypical symptom that cannot be explained as an effect of RYGB should be considered a possible indicator of TB and thoroughly investigated. Once the diagnosis is confirmed, TB should be treated in a similar fashion like all other patients, with no drug modifications needed. TB has a good overall prognosis in a compliant patient. We recommend raising awareness in the healthcare profession about this rare complication of RYGB in the need of time.
